# The Phylogenomic Characterization of *Planotetraspora* Species and Their Cellulases for Biotechnological Applications

**DOI:** 10.3390/genes15091202

**Published:** 2024-09-12

**Authors:** Noureddine Bouras, Mahfoud Bakli, Guendouz Dif, Slim Smaoui, Laura Șmuleac, Raul Paşcalău, Esther Menendez, Imen Nouioui

**Affiliations:** 1 Laboratoire de Valorisation et Conservation des Ecosystèmes Arides (LVCEA), Faculté des Sciences de la Nature et de la Vie et Sciences de la Terre, Université de Ghardaia, B.P. 455, Ghardaïa 47000, Algeria; noureddine_bouras@yahoo.fr; 2 Laboratoire de Biologie des Systèmes Microbiens (LBSM), Ecole Normale Supérieure de Kouba, Algiers 16308, Algeria; kdgb2007@yahoo.fr; 3 Département des Sciences Naturelles, École Normale Supérieure de Laghouat, B.P. 4033, Laghouat 03000, Algeria; 4 Laboratory of Microbial Biotechnology and Engineering Enzymes (LMBEE), Center of Biotechnology of Sfax (CBS), University of Sfax, Road of Sidi Mansour Km 6, P.O. Box 1177, Sfax 3018, Tunisia; slim.smaoui@yahoo.fr; 5 Faculty of Agriculture, University of Life Sciences “King Mihai I” from Timişoara, 119 Calea Aradului, 300645 Timişoara, Romania; raulpascalau@yahoo.com; 6 Departamento de Microbiología y Genética, Instituto de Investigación en Agrobiotecnología (CIALE), Universidad de Salamanca, 37008 Salamanca, Spain; esthermenendez@usal.es; 7 Leibniz Institute, DSMZ-German Collection of Microorganisms and Cell Cultures GmbH, 38124 Braunschweig, Germany; ino20@dsmz.de

**Keywords:** *Planotetraspora*, in silico study, genomic, metabolomics, cellulase, 3D structure, molecular docking

## Abstract

This study aims to evaluate the in silico genomic characteristics of five species of the genus *Planotetraspora*: *P. kaengkrachanensis*, *P. mira*, *P. phitsanulokensis*, *P. silvatica*, and *P. thailandica*, with a view to their application in therapeutic research. The 16S rRNA comparison indicated that these species were phylogenetically distinct. Pairwise comparisons of digital DNA-DNA hybridization (dDDH) and OrthoANI values between these studied type strains indicated that dDDH values were below 62.5%, while OrthoANI values were lower than 95.3%, suggesting that the five species represent distinct genomospecies. These results were consistent with the phylogenomic study based on core genes and the pangenome analysis of these five species within the genus *Planotetraspora*. However, the genome annotation showed some differences between these species, such as variations in the number of subsystem category distributions across whole genomes (ranging between 1979 and 2024). Additionally, the number of CAZYme (Carbohydrate-Active enZYme) genes ranged between 298 and 325, highlighting the potential of these bacteria for therapeutic research applications. The in silico physico-chemical characteristics of cellulases from *Planotetraspora* species were analyzed. Their 3D structure was modeled, refined, and validated. A molecular docking analysis of this cellulase protein structural model was conducted with cellobiose, cellotetraose, laminaribiose, carboxymethyl cellulose, glucose, and xylose ligand. Our study revealed significant interaction between the *Planotetraspora* cellulase and cellotetraose substrate, evidenced by stable binding energies. This suggests that this bacterial enzyme holds great potential for utilizing cellotetraose as a substrate in various applications. This study enriches our understanding of the potential applications of *Planotetraspora* species in therapeutic research.

## 1. Introduction

The genus *Planotetraspora* was first described by Runmao et al. [1] as a member of the family *Streptosporangiaceae*, displaying distinct characteristics [2]. At the time of writing, the genus *Planotetraspora* comprises only five type strains (https://lpsn.dsmz.de/genus/planotetraspora (accessed on 1 June 2024)). These species are *P. mira* [1], *P. silvatica* [3], *P. thailandica* [3], *P. kaengkrachanensis,* and *P. phitsanulokensis* [4]. The members of this genus exhibit specific characteristics in their cell wall peptidoglycan and whole-cell composition [5,6]. *P. mira* (NA9211028^T^ = ATCC 51423^T^ = DSM 44359^T^ = JCM 9131^T^ = NBRC 15435^T^) was isolated from a soil sample collected in Sichuan Province, People’s Republic of China [1]. However, *P. silvatica* (TT 00-51^T^ = DSM 44746^T^ = JCM 12867^T^ = NBRC 100141^T^) was isolated from the soil of Amami Island, Japan [3]. *P. thailandica* (BCC 21825^T^ = DSM 45400^T^ = JCM 16140^T^ = NBRC 104271^T^) was collected near a hot spring in Krabi Province, Thailand [4]. In addition, *P. kaengkrachanensis* (A-T 0875^T^ = BCC 24832^T^ = NBRC 104272^T^) and *P. phitsanulokensis* (A-T 1383^T^ = BCC 26045^T^ = NBRC 104273^T^) were isolated from Prachuap Khiri Khan Province and Nakhon Thai District in Phitsanulok Province, Thailand, respectively [2]. Some *Planotetraspora* strains showed antibacterial activity, antifungal activity, or LC-MS spectra, suggesting secondary metabolites with PKS and NRPS genes detected for biosynthetic potential [7]. These results suggest that *Planotetraspora* species possess significant biosynthetic potential [7]. No other study has been published on the bioactive compounds of this genus. The genus *Planotetraspora* exhibits an intriguing ability to thrive in various pedological environments [1,2,3,4]. Additionally, this investigation unveils a repertoire of cellulase genes within these bacterial strains. This diversity in cellulolytic enzymes suggests promising biotechnological applications and prompts further exploration into their enzymatic capabilities. Further research is focused on elucidating their cellulolytic machinery.

Cellulase enzymes, including endoglucanases (EC 3.2.1.4), β-glucosidase (EC 3.2.1.21), and exoglucanases (EC 3.2.1.91), play a key role in breaking down cellulose by hydrolyzing β-1,4-glycosidic bonds to produce cello-oligosaccharides [8]. These enzymes are essential for the bioconversion of cellulosic biomass. Derived from renewable resources and waste, they are invaluable in industries such as biofuels, textiles, paper, laundry, agriculture, food, and beverages [9]. Their wide range of applications also extends to detergents and other biotechnology fields, with increasing focus on their role in the textile sector [10]. Additionally, ongoing advancements have been made in developing new strategies to boost the production of microbial cellulases. These strategies include using waste feedstock as a substrate for producing individual enzymes or enzyme cocktails, controlling process parameters, and implementing genetic modifications to improve enzyme yield, efficiency, and specificity [11]. Over the past few decades, several bioinformatics tools have been developed to elucidate the functional properties of cellulase enzymes at the molecular level [9].

The aim of this study is to analyze the genome and carbohydrate-active enzymes of the *Planotetraspora* species through in silico profiling. Furthermore, a computational approach was conducted for understanding the functional and structural properties of *Planotetraspora* cellulases.

## 2. Materials and Methods

### 2.1. Genomic Dataset

We performed taxonomic analyses using genome data and applied various bioinformatics methods to evaluate the relationships among different species of *Planotetraspora*. The genome sequences of known *Planotetraspora* species were obtained from the GenBank database. In addition, we utilized DFAST (https://dfast.ddbj.nig.ac.jp/, accessed on 8 September 2024) to identify G + C content (%), the number of CDSs (Coding DNA Sequences), rRNA, tRNA, and CRISPR regions (which are sequences originating from DNA fragments of bacteriophages). All data analyzed during this study (genome assembly, GenBank assembly accession number, etc.) are available through the NCBI GenBank database (http://www.ncbi.nlm.nih.gov, accessed on 8 September 2024). A summary of the genome sequence characteristics is provided and can be accessed using the accession numbers listed in Table 1.

### 2.2. Phylogenetic and Phylogenomic Analyses

The 16S rRNA gene sequences of each species of *Planotetraspora* were compared to calculate the percentage of similarity with closely related type strains found in the EzBioCloud database (https://www.ezbiocloud.net, accessed on 8 September 2024) [12]. The digital DNA-DNA hybridization (dDDH) values and confidence intervals were then calculated with 100 replicates, following the recommended parameters of the Genome-to-Genome Distance Calculator (GGDC; http://ggdc.dsmz.de, accessed on 8 September 2024) [13,14]. Pairwise genome comparison between genomes of different species of *Planotetraspora* was assessed by calculating the average nucleotide identity (OrthoANI) using EzGenome database (https://www.ezbiocloud.net/tools/ani (accessed on 3 June 2024)). [15]. Phylogenomic analyses were performed through the Type Strain Genome Server (TYGS, https://tygs.dsmz.de/, accessed on 8 September 2024) [16]. A genome-based phylogenetic tree was constructed using FastME [17] from the Genome Blast Distance Phylogeny (GBDP). The tree was rooted at the midpoint [18] and visualized with PhyD3 [19]. Branch supports were inferred from 100 pseudo-bootstrap replicates.

### 2.3. Core Gene and Pangenome Analysis

The core genes present in all *Planotetraspora* species were identified using Roary version 3.13.0 [20] with default settings. The MEGA software version 11 [21] was also used, with 1000 bootstrap replications to assess statistical support. This analysis was conducted using the GFF3 files of the selected genomes, which were annotated previously [22]. The maximum likelihood phylogenetic tree produced by Roary was then visualized using the Interactive Tree of Life (iToL) [23]. To represent the presence or absence of each core gene in every strain, Phandango [24] was utilized. The proportions of the pangenome were visualized using the summary statistics file generated by Roary.

### 2.4. Genome Annotation

Gene prediction and annotation of the whole genome sequences was carried out by using the Rapid Annotations via the Subsystems Technology (RASTtk pipeline v.2.0, https://rast.nmpdr.org/, accessed on 8 September 2024) server [25].

### 2.5. Carbohydrate-Active Enzymes (CAZyme)

The dbCAN3 database (http://bcb.unl.edu/dbCAN2/ (accessed on 4 June 2024), [26]) was used to determine the abundances and distributions of clusters of different families of CAZymes (Carbohydrate-Active enZYmes) and their associated domains in each of the studied genomes.

### 2.6. Sequence Retrieval

Cellulase (named cellulase family glycosylhydrolase) protein sequences originating from different *Planotetraspora* were obtained from the NCBI database (http://www.ncbi.nlm.nih.gov, accessed on 8 September 2024). The sequences retrieved in FASTA format from *Planotetraspora* spp. included: *P. phitsanulokensis*, accession number: WP_204072101; *P. silvatica*, accession number: WP_203975166; *P. mira*, accession number: WP_203952344; *P. thailandica*, accession number: WP_203947776; and *P. kaengkrachanensis*, accession number: WP_203882566.

### 2.7. In Silico Physico-Chemical Properties

The ExPASy Prot Param (https://web.expasy.org/protparam/ (accessed on 5 June 2024)) server tool was utilized to determine the in silico physico-chemical properties of the cellulase protein sequences [27]. Our analysis encompassed parameters like molecular weight, theoretical isoelectric point (pI), the number of positively (+R) and negatively (−R) charged amino acid residues, extinction coefficient (EC), instability index (II), aliphatic index (AI), and grand average of hydropathicity (GRAVY).

### 2.8. Homology Modeling and Model Confirmation

There is no available three-dimensional structure for cellulase from *Planotetraspora* spp., and it was therefore essential to employ homology modeling methodology. This approach predicts the protein’s three-dimensional structure by utilizing template sequences with known structures. Subsequently, the SWISS-MODEL server (http://swissmodel.expasy.org/, accessed on 8 September 2024) [28] was used to generate the 3D models of the target proteins. The derived homology structural model was refined for energy minimization using an algorithm for high-resolution protein structure refinement, the ModRefiner server (http://zhanglab.ccmb.med.umich.edu/ModRefiner/ (accessed on 5 June 2024)) [29]. The structural model’s quality was evaluated by examining stereo-chemical parameters of the refined via PROCHECK tool on the SAVES v6.0 online server (https://saves.mbi.ucla.edu/, accessed on 8 September 2024) [30]. The results were viewed and optimized by PyMol software, version 2.3 [31].

### 2.9. Molecular Docking Analysis

For the selection of ligands in the molecular docking study, the PubChem database (https://pubchem.ncbi.nlm.nih.gov/ (accessed on 15 June 2024)) was employed. Consequently, four polysaccharide ligands (cellobiose, cellotetraose, laminaribiose, and carboxymethyl cellulose) along with two monosaccharide ligands (glucose and xylose) were chosen.

The ligands were retrieved in SDF file format and converted to PDB format using the PyMol tool, version 2.3. Before protein–ligand docking with CB-DOCK, the energy of all ligands was minimized using Chem3D Ultra 8.0 software. The CB-Dock server (http://clab.labshare.cn/cb-dock/php/index.php (accessed on 15 June 2024)), in collaboration with AutoDock Vina, employs a curvature-based cavity detection method to predict protein binding sites, achieving a success rate exceeding 70% [32]. This molecular docking analysis comprised the refined cellulase A2 enzyme file and the minimized ligands in PDB format. It aimed to identify five potential binding cavities and explore diverse enzyme–ligand interaction models. Ultimately, the model with the lowest binding energy, determined by the lowest Vina value, was selected. Visualization of ligand-binding amino acid residues with the receptor was performed using BIOVIA Discovery Studio Visualizer.

## 3. Results and Discussion

### 3.1. Phylogenetic and Phylogenomic Analyses

**Figure 1:**
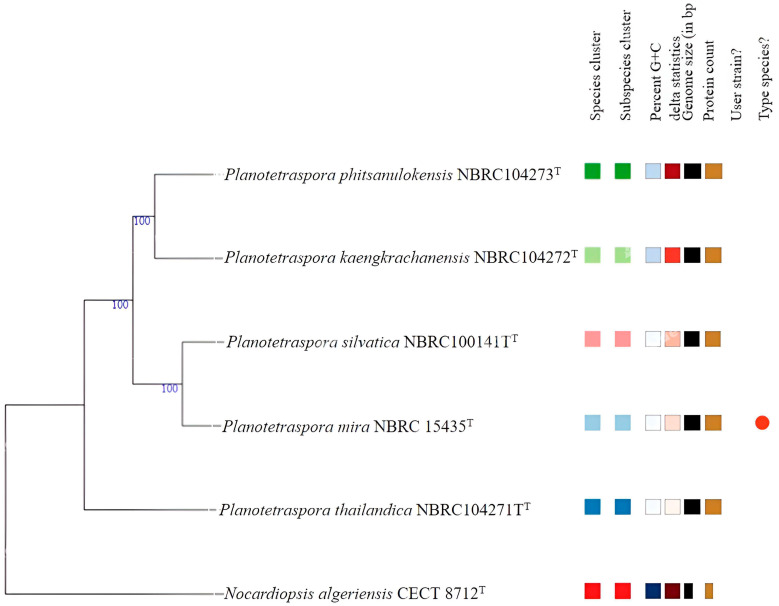
Phylogenomic tree based on genome sequences in the TYGS tree inferred with FastME 2.1.6.1 [16] from the Genome BLAST Distance Phylogeny approach (GBDP); distances calculated from genome sequences. The branch lengths are scaled in terms of GBDP distance formula d5. The numbers above branches are GBDP pseudo-bootstrap support values >70% from 100 replications. The tree was rooted at the midpoint [13]. The different color indicates the different species cluster.

The general features of *Planotetraspora* species are recorded in Table 1. Based on DFAST, the digital DNA G + C content ranged between 69.2 and 69.7%. However, the NCBI database showed values of 69.0 to 69.5%. All genomic features, number of predicted protein-coding gene sequences (CDSs), number of rRNA, tRNA, and CRISPRs are summarized in Table 1. These data indicated that the five species of *Planotetraspora* are genetically very close. This finding is supported by the obtained values of 16S rRNA indicated values from 98.27 (between *P. mira* and *P. phitsanulokensis*) to 99.65% (between *P. mira* and *P. silvatica*). On the other hand, pairwise dDDH and OrthoANI values between these studied species were ranged, respectively, between 28.8 and 62.3%, and between 84.47 and 95.25% (Table S1). Considering the recent recommendations for dDDH (70%) and OrthoANI (95–96%) threshold values [15,33] for bacterial species delineation, the five species represent distinct genomospecies. These findings were consistent with the phylogenomic positioning of these species within the genus *Planotetraspora* (Figure 1).

### 3.2. Core Gene and Pangenome Analysis

**Figure 2:**
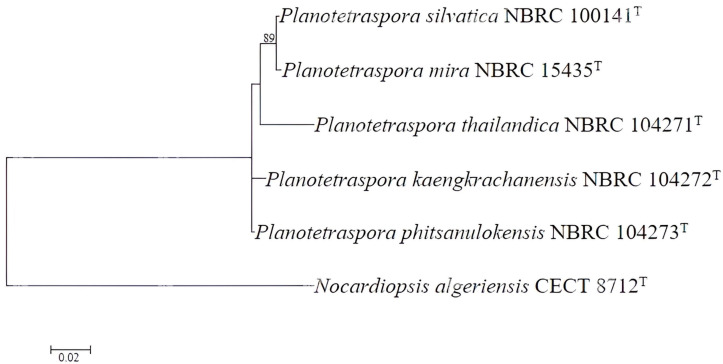
Maximum likelihood core gene phylogenomic tree. The core genes were identified using the Roary program [20] and MEGA software version 11 [21] with 1000 bootstrap replications to assess statistical support. This illustrates the evolutionary relationship between the species of *Planotetraspora*. *Nocardiopsis algeriensis* CECT 8712^T^ was used as an outgroup. Bar 0.02 nucleotide substitution per site.

Core genes are genes that are present in all members of a particular bacterial group. These genes are often used to determine the evolutionary relationships between different species and can help in understanding the genetic diversity and relatedness within a genus. By analyzing core genes, it is possible to classify bacterial species more accurately and understand their evolutionary history. In our case, the maximum likelihood core gene tree consistently demonstrated that all species of *Planotetraspora* are closely related, but as separate species, as indicated by the clustering of these species in the core gene phylogenomic tree (Figure 2).

**Figure 3:**
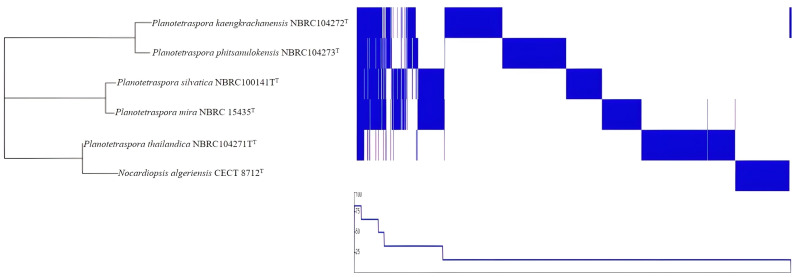
Pangenome analysis of five species of genomes of *Planotetraspora* was conducted using Roary [20]. Matrix destitution of genes across the pangenome of all *Planotetraspora* species (from Rotary), and *Nocardiopsis algeriensis* CECT 8712^T^ was used as an outgroup. Pangenome visualization is displayed as presence (blue) and absence (white) output using Phandango [24].

Pangenome analysis is based on the core and accessory genomes and is of great interest because it allows us to study the genetic diversity and evolution of bacterial species at a genomic level. By analyzing the pangenome, which consists of the core genome and the accessory genome, scientists can better understand the genetic diversity within a bacterial species. This information is helpful in bacterial taxonomy. In essence, pangenome analysis provides a more comprehensive view of bacterial diversity and evolution than traditional methods based on a single reference genome. The pangenome analysis of *Planotetraspora* species was performed in Roary program. A summary of the pangenome composition provided by Roary was studied. The pangenome of four strains contains 29,377 genes, of which the core genome represents only 1.9% (557 genes) and the shell genome represents 98.1% (28820 genes), constituting the largest part of the gene pool among these six species. The pangenome visualization of the five *Planotetraspora* species (Figure 3) was generated by uploading Roary’s gene presence/absence matrix to Phandango.

### 3.3. Genome Annotation

The RASTtk Model SEED database was used. Annotation revealed that 16% of the predicted coding sequences (CDSs) were assigned with subsystems (the same value for all *Planotetraspora* species). The most represented subsystems among the five draft genomes were carbohydrates and amino acids and their derivatives. Other subsystems that were common were associated with protein metabolism, cofactors, vitamins, prosthetic groups, pigments, fatty acids, lipids, and isoprenoids. The numbers of annotated genes classified according to the subsystems are listed in Table S2.

### 3.4. Carbohydrate-Active Enzymes (CAZymes) and Cazome

The server dbCAN3 integrates three tools for CAZyme annotation: HMMER, DIAMOND, and Hotpep. HMMER uses Hidden Markov Models (HMMs) to identify CAZymes based on their sequence profiles (derived from eCAMI classification of CAZyDB families). DIAMOND performs sequence alignment to compare sequences against a database of known CAZymes using a BLAST-like approach. Hotpep identifies conserved short peptides within CAZyme sequences. A higher “Tools” value indicates how many of these tools have independently confirmed the presence of a CAZyme family in the sequence, thereby increasing the confidence in the annotation, as it means multiple independent methods have corroborated the presence of the CAZyme family. The CAZyme family is annotated only when it has been detected by all three bioinformatics tools.

CAZymes are involved in the synthesis, modification, or breakdown of carbohydrates (sugars). Analyses using the dbCAN3 database identified 195 to 228 CAZymes in the five *Planotetraspora* species (Table 2). These enzymes constitute between 2.34% and 2.70% of protein-coding genes, forming what is known as the Cazome. These values align with the estimated range for CAZyme-encoding genes in all microorganism genomes [34,35]. By studying the cazome, scientists can understand the metabolic capabilities of these microorganisms, such as the types of carbohydrates they can utilize and the types of sugars they can produce.

The exploration of CAZyme diversity unveiled various enzymatic families, including glycosyl transferases (GTs), glycosyl hydrolases (GHs), Carbohydrate Esterases (CEs), Carbohydrate-Binding domains (CBMs), Polysaccharide Lyases (PLs), and enzymes with auxiliary activities (AAs), as reported in Table 2.

The most abundant enzymatic family predicted in the studied genomes was glycosyl transferases, with GT4 (22 to 30 CAZymes) and GT2 (15 to 20 CAZymes) encoding genes for each species. Glycosyl transferases (EC 2.4) catalyze the transfer of a sugar molecule from one molecule to another, playing a crucial role in the biosynthesis of complex carbohydrates, glycoproteins, and glycolipids [34]. These enzymes are essential for various biological processes, including cell–cell recognition, cell signaling, and immune response modulation. The CAZy analysis revealed 46 different sub-categories of glycoside hydrolases. Glycosyl hydrolases are involved in a wide range of biological processes, including the degradation of plant cell walls by microorganisms. These enzymes play a crucial role in breaking down complex carbohydrates into simpler sugars, which can then serve as a source of energy for the microorganism. Glycosyl hydrolases catalyze the hydrolysis of glycosidic bonds within complex carbohydrates like starch, glycogen, and cellulose. For instance, cellulase, which is a type of glycoside hydrolase enzyme that plays a crucial role in the breakdown of cellulose into smaller sugar molecules.

Sharada et al. [36] underscored the importance of cellulases, highlighting their diverse applications across various industries, including human food (coffee processing, wine making, and fruit juice production), healthcare, beverages, animal feed, agriculture, brewing, dairy products, laundry detergents, textiles, pharmaceuticals, waste treatment, and the paper and pulp industry. These enzymes play a crucial role in bioconversion, converting agricultural waste into sugar and bioethanol [37]. Notably, cellulase is gaining recognition as an effective alternative to available antibiotics for treating biofilms produced by *Pseudomonas*, presenting a promising trend to address antibiotic-resistant bacteria in the healthcare sector [38].

Microbial cellulases, known for their broad applicability and cost-effectiveness, are increasingly popular in various industries. While cellulases have demonstrated promise in health and medicine, contributing to the treatment of conditions like phytobezoars disease and combating pathogenic biofilms, further studies are necessary for a comprehensive understanding of their potential in these fields [39,40]. A notable potential application of cellulase in medicine involves the degradation of cell walls in pathogenic organisms. For example, *Acanthamoeba*, a Protista that causes a rare and severe corneal infection known as keratitis, can lead to blindness. Cellulases could be utilized to break down the cyst wall and control the pathogen, offering a potential avenue for treatment; however, extensive research is necessary before considering cellulase as an eye treatment [41,42]. Pathogenic microorganisms often form biofilms, which are microbial cell assemblages irreversibly associated with surfaces and enclosed in an extracellular polymeric substance (EPS) matrix. These biofilms are commonly found on various surfaces, including living tissues and indwelling medical devices such as artificial hip prostheses, central venous catheters, prosthetic heart valves, intrauterine devices, and urinary catheters. Cellulases exhibit potential for efficiently removing such biofilms from medical devices [37]. Further research is required to explore the effectiveness of cellulases in this application.

### 3.5. Sequence Retrieval and Physico-Chemical Properties

The cellulase from *Planotetraspora* spp., cellulase glycosylhydrolase family, was retrieved from the NCBI database and is also available in the UniProt database (https://www.uniprot.org/) under the name glycoside hydrolase family 5 domain-containing protein. The physico-chemical feature data on *Planotetraspora* spp. cellulases encompassed their pI, size, ratio of negatively charged versus positively charged amino acids, extinction coefficient (EC), instability index (II), aliphatic index (AI), and grand average of hydropathicity (GRAVY) (Table 3).

The cellulase proteins displayed a pI below 7, suggesting an acidic nature. The protein displayed a higher number of negatively charged residues compared to positively charged ones. Maintaining protein stability is essential for various biological processes. According to the Beer–Lambert law, the extinction coefficient (EC) quantifies light absorption by a protein at a given wavelength [43]. Hence, the extinction coefficient for the target cellulases ranged from 92,040 to 93,530. Protein stability is often assessed using the instability index (II). A II below 40 indicates stability, while a II above 40 suggests instability [27]. All *Planotetraspora* cellulases have an index of instability below 40, reflecting their stability.

Moreover, the aliphatic index (AI) provides another measure of protein stability, with previous research indicating a positive relationship between AI and protein thermostability [44]. The enzyme’s aliphatic index (AI), which reflects the volume occupied by the side chains of Ala, Val, Leu, and Ile amino acids, was found to exceed 70 for the cellulases of *Planotetraspora* species. This suggests a high level of thermostability for these proteins. Each amino acid hydropathy value in the query sequence is computed and divided by the total number of residues to obtain the protein’s GRAVY score. The resulting values for the cellulases are negative, indicating their hydrophilic nature.

### 3.6. Homology Modeling and Model Confirmation

**Figure 4:**
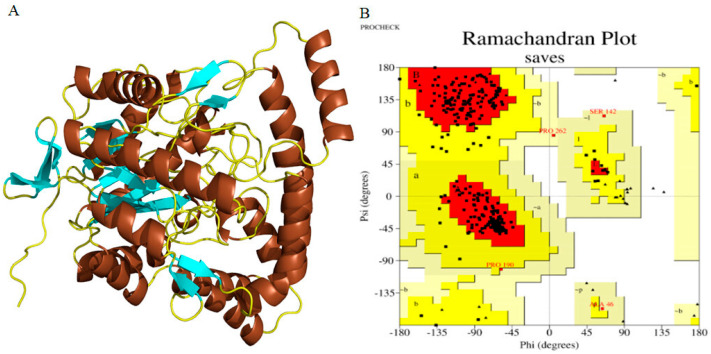
The SWISS−MODEL generated the 3D and refined structure of *Planotetraspora* cellulase enzymes and its validation. (**A**) This structural model was refined by ModRefiner and visualized by PyMOL. α-helix (chocolate), β-sheet (cyan), and loop (yellow). (**B**) Ramachandran plot for the refined cellulase obtained by PROCHECK. The residues found in favored (A, B, and L), additional allowed (a, b, l, and p), generously allowed (~a, ~b, ~l, and ~p), and disallowed regions are delineated with red, yellow, beige, and white color coding, respectively. All non-glycine and non-proline residues are depicted as filled black squares, while glycines (non−terminal) are represented as filled black triangles.

The amino acid sequences of cellulases from *Planotetraspora* species were subjected to homology modeling via the SWISS-MODEL web server to generate the 3D structures. The aryl-phospho-β-D-glucosidase BglC, GH1 family protein corresponding to the A0A239AH65_9ACTN model in AlphaFold DB from *Streptosporangium subroseum*, was utilized as a template to perform the homology modeling of the target cellulases (Figure 4A). Furthermore, the model’s quality was evaluated using PROCHECK server’s stereochemical parameters. An energy-minimized Ramachandran plot for both initial and refined cellulase structures was generated, with the *x*-axis representing Phi angles and the *y*-axis representing Psi angles. The plot is divided into four quadrants: most favored (91.5%), additional allowed (8.0%), generously allowed (0.5%), and disallowed regions (0.0%) (Figure 4B).

### 3.7. Molecular Docking Analysis

The analysis of molecular docking involved exploring potential interactions between ligands and the target *Planotetraspora* cellulase in enzyme catalysis. Docking for all ligands and the refined cellulase was carried out using the CB-Dock server among the six potential ligands (Table 4).

The table included cellobiose, cellotetraose, laminaribiose, carboxymethyl cellulose, glucose, and xylose; the cellotetraose ligand (PubChem CID, 439626) was selected due to its lowest binding energy with the cellulase receptor, recorded at −8.3 kJ/mol (Table 5).

**Figure 5:**
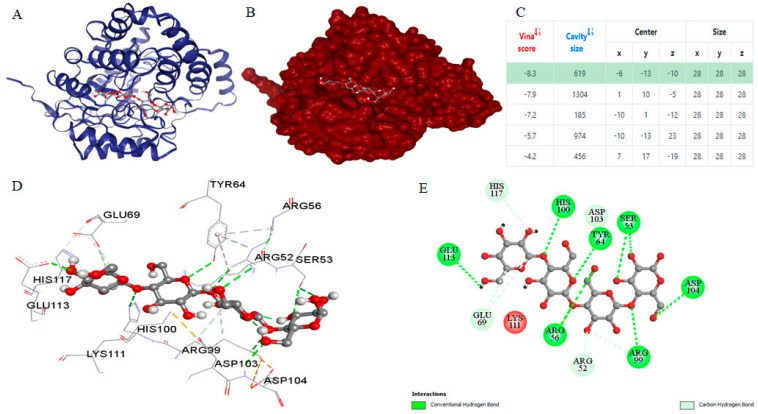
Molecular docking analysis of the cellotetraose ligand with the *Planotetraspora* cellulase enzyme receptor. (**A**) The cartoon representation of the protein receptor and its ligand. (**B**) Cavity illustration showed the region on the protein receptor surface where bonding occurs with the ligand. (**C**) Vina score and cavity size models table displayed the Vina score of this receptor and the ligand docking with the best highlighted score. The enzyme–ligand interactions were visualized, employing the ball and stick style for the ligand and the surface style for the receptor. Ligand and receptor colors were configured based on elements and B-factor, respectively. (**D**) Interaction residues depicted the interacting residues of the receptor with the ligand as visualized in BIOVIA Discovery Studio Visualizer. (**E**) 2D diagram of residue interaction types between ligand and receptor as visualized in BIOVIA Discovery Studio Visualizer.

The results obtained through CB-DOCK for the most suitable ligand, identified by its lowest binding energy according to Vina scores and estimated cavity size, are depicted in Figure 5. Employing the curvature-dependent surface area model [45], five potential interaction models were established to predict the binding affinity between the *Planotetraspora* cellulase enzyme model and the selected ligands. Furthermore, the molecular docking visualization results clearly demonstrate that the cellotetraose ligand engages with specific residues within the cellulase A2 enzyme receptor. These residues include Arg52, Ser53, Arg56, Tyr64, Glu69, Arg99, His100, Asp103, Asp104, Glu113, and His117. Notably, a significant proportion of these interacting residues participate in establishing crucial hydrogen bonds (H-bonds) with the cellotetraose substrate. This observation underscores the importance of these interactions in facilitating the binding and catalytic activity of the enzyme model.

This study is consistent with the molecular docking analysis of a cellulase from *Acinetobacter* sp. showing that the interactions of cellotetraose is favored by Leu156, Ala157, and Val213 by forming H bonds and that cellulase has the greater potential towards the cellotetraose as a substrate for the high yield of ethanol [46]. Similarly, in a study by Khairudin and Mazlan [47], catalytic interactions of β-glucosidase from *Paenibacillus polymyxa* with its substrates including cellobiose, cellotetraose, and cellotetriose were investigated using molecular docking. Their findings indicated that cellobiose, cellotetraose, and cellotetriose exhibited binding affinities with docking scores of −6.2 kJ/mol, −5.68 kJ/mol, and −5.63 kJ/mol, respectively [47]. Therefore, these results could offer valuable insights for designing more efficient hydrolyzing enzymes. This study implies that modifying residues within the catalytic site can improve cellulase’s enzymatic hydrolysis activity, leading to higher yields and potentially reducing production costs [46]. In addition, these findings could have significant implications for enhancing bacterial cellulase efficiency. Cellulase is a key enzyme in the global market due to the annual disposal of nearly half a billion tons of cellulosic waste in the U.S. [48,49]. Microbial cellulases have widespread applications in the textile, pulp, brewing, food, and agriculture industries [50]. Additionally, cellulases are essential for carotenoid extraction, pharmaceuticals, genetic engineering, and pollution treatment [36]. Enhancing cellulase efficiency promotes sustainable waste conversion into valuable resources such as sugars and bioethanol. Improvements in enzymatic hydrolysis activity through modifications in catalytic site residues positively impact industries like animal feed, textiles, paper production, biofuel production, genetic engineering, and pollution treatment [51,52,53,54,55]. Additionally, cellulase supplements are commonly used to aid the digestion of plant-based foods by breaking down cellulose, a major component of dietary fiber that human enzymes cannot digest. This aids in improving digestion and nutrient absorption, and is particularly beneficial for individuals with conditions such as bloating, gas, or constipation. Enhancing nutrient absorption can alleviate symptoms of digestive disorders, thereby improving overall digestive health [56]. In the pulp and paper industry, cellulases are used for pulping, refining, and deinking [57,58,59]. Recent studies show that cellulase treatment enhances fiber porosity and reactivity in the viscose rayon process [60].

## 4. Conclusions

This present report compared phylogenetic and phylogenomic analyses, core gene and pangenome analysis, genome annotation, and carbohydrate-active enzymes to evaluate the potential of these strains for therapeutic research applications. Additionally, we investigated the in silico physico-chemical characteristics of cellulases from *Planotetraspora* species, illuminating their potential roles in therapeutic research alongside their genomic profiles. In silico physico-chemical characterization, structural homology modeling, refinement, and validation were performed for the cellulase from *Planotetraspora* species. Substrate specificity was assessed through molecular docking analysis, revealing that, among the tested polysaccharide and monosaccharide ligands, cellotetraose exhibited the highest activity against the cellulase, with a docking score of −8.3 kJ/mol. Our in silico analysis offers insights for experiments aiming to design more efficient cellulose enzymes for enzymatic hydrolysis processes, potentially increasing yields and reducing production costs.

## Figures and Tables

**Table 1:** Features of the genome sequences obtained by using DFAST server.

	*Pk*	*Pm*	*Pp*	*Ps*	*Pt*	*Na*
**Genome assembly**	ASM1686289v1	ASM1686327v1	ASM1686329v1	ASM1686331v1	ASM1686333v1	ASM1420369v1
**GenBank assembly accession number**	GCA_016862895.1	GCA_016863275.1	GCA_016863295.1	GCA_016863315.1	GCA_016863335.1	GCA_014203695.1
**Total length (bp)**	8,768,202	9,027,692	9,167,700	8,664,980	8,961,068	4,812,129
**GC content (%)**	69.7 (69.5 ^a^)	69.3 (69.5 ^a^)	69.6 (69.5 ^a^)	69.3 (69.5 ^a^)	69.2 (69.0 ^a^)	71.3 (71.5 ^a^)
**Gap Ratio (%)**	0.0%	0.0%	0.0%	0.0%	0.0%	0.017684%
**No. of CDSs**	8191	8439	8579	8033	8304	4265
**No. of rRNA**	3	2	2	1	2	2
**No. of tRNA**	75	69	72	72	76	65
**No. of CRISPRS**	4	15	3	18	64	6
**Coding ratio (%)**	88.2%	88.1%	88.0%	87.6%	87.7%	86.5%

*Pk*, *P. kaengkrachanensis*; *Pm*, *P. mira*; *Pp*, *P. phitsanulokensis*; *Ps*, *P. silvatica*; *Pt*, *P. thailandica*; *Na*, *Nocardiopsis algeriensis*.^a^: values obtained by using NCBI.

**Table 2:** Comparison of predicted CAZymes in five *Planotetraspora* species with the CAZy database using dbCAN3 tools (HMMER, DIAMOND, and Hotpep).

	*Pk*	*Pm*	*Pp*	*Ps*	*Pt*
**AA3**	2	1	1	1	1
**AA10 + CBM12**	1	0	0	0	0
**CBM2|GH6**	0	1	0	0	0
**CBM2|GH18**	2	3	3	3	2
**CBM3|GH0**	1	1	1	1	1
**CBM5|GH18**	0	0	1	0	1
**CBM6**	5	4	5	3	5
**CBM6|GH3**	0	2	2	1	0
**CBM6|GH99**	1	1	1	1	0
**CBM13**	1	1	1	1	0
**CBM13 + CBM6**	1	0	0	0	0
**CBM13 + CBM92**	0	0	0	1	0
**CBM13|GH18**	1	0	1	1	1
**CBM13|GH30**	1	1	1	1	1
**CBM13|GH39**	2	2	2	0	0
**CBM13|GH55**	1	1	0	1	1
**CBM13|GH141**	1	0	0	0	0
**CBM16|GH18**	2	2	2	2	3
**CBM32**	6	6	7	6	6
**CBM32|CBM6**	1	1	1	1	1
**CBM32|GH2**	2	2	2	1	1
**CBM32|GH3**	0	0	0	0	1
**CBM32|GH16**	1	2	2	2	2
**CBM32|GH20**	1	1	1	1	1
**CBM32|GH28|GH29**	1	1	0	0	0
**CBM32|GH29**	2	1	2	1	3
**CBM32|GH46**	1	1	1	1	0
**CBM32|GH55**	1	1	1	1	1
**CBM32|GH85**	1	1	1	0	1
**CBM32|GH87**	3	3	3	3	3
**CBM32|GH92**	1	1	1	1	1
**CBM32|GH95**	0	1	0	1	1
**CBM32|GH99**	1	1	1	1	0
**CBM32|GH120|GH95**	0	0	1	0	1
**CBM32|GH141**	1	1	0	1	1
**CBM32|GH158|GH16**	1	1	1	1	0
**CBM35|GH2**	1	1	1	1	0
**CBM35|GH20**	1	1	1	0	0
**CBM35|GH27**	0	0	1	0	1
**CBM35|GH75**	1	1	1	1	1
**CBM48|GH13**	4	4	4	4	4
**CBM51|GH27**	1	1	1	1	1
**CBM51|GH97**	1	1	1	1	1
**CBM57**	0	1	0	0	1
**CBM57|GH18**	1	1	1	1	0
**CBM61|GH53**	0	1	1	1	0
**CBM67|GH78**	0	0	0	0	1
**CBM92**	1	1	1	1	1
**CE0**	1	1	1	0	0
**CE1**	1	1	1	1	1
**CE4|GT2**	1	1	1	1	1
**CE7**	2	2	2	2	2
**CE9**	1	1	1	1	1
**CE14**	3	3	3	3	3
**GH0**	2	2	2	2	1
**GH1**	7	8	7	6	6
**GH2**	4	5	5	3	5
**GH3**	9	9	9	8	6
**GH4**	3	3	3	3	3
**GH5**	2	2	2	2	2
**GH6**	0	0	0	0	1
**GH9**	1	1	1	0	0
**GH13**	8	8	8	8	8
**GH15**	3	2	2	3	3
**GH18**	2	1	2	1	1
**GH20**	5	5	5	4	4
**GH23**	2	2	2	2	2
**GH27**	1	1	1	1	0
**GH29**	0	1	0	0	0
**GH31**	2	3	2	3	0
**GH33**	0	1	1	0	0
**GH35**	1	1	1	1	2
**GH36**	4	3	3	3	3
**GH38**	6	6	6	6	5
**GH42**	0	2	1	2	0
**GH43**	1	1	2	1	1
**GH46**	0	0	0	0	1
**GH50**	0	2	0	0	0
**GH51**	1	2	1	1	0
**GH55**	1	1	1	1	1
**GH63**	1	1	1	0	0
**GH65**	1	1	1	1	1
**GH78**	0	1	0	0	3
**GH87**	1	1	1	1	1
**GH92**	1	1	2	1	2
**GH93**	0	0	0	1	0
**GH95**	2	2	2	1	2
**GH99**	0	0	0	0	1
**GH106**	0	1	0	0	3
**GH110**	2	1	1	1	0
**GH114**	1	1	1	1	0
**GH121**	2	1	2	1	1
**GH127**	2	2	2	1	1
**GH128**	1	1	1	1	1
**GH130**	0	1	0	1	0
**GH141**	0	0	0	0	1
**GH146**	0	1	2	1	1
**GH151**	0	1	0	0	0
**GH154**	1	1	1	1	1
**GH171**	2	2	2	1	1
**GT0|GT2**	0	0	0	1	0
**GT1**	4	4	5	5	5
**GT2**	18	17	20	17	15
**GT4**	26	30	30	30	22
**GT8**	0	0	0	0	1
**GT9**	1	1	1	1	1
**GT20**	2	2	2	2	2
**GT28**	2	2	2	2	3
**GT35**	1	1	1	1	1
**GT39**	3	3	3	3	2
**GT51**	5	5	5	5	5
**GT81**	1	1	1	1	1
**GT83**	1	1	1	1	1
**GT87**	1	1	1	1	1
**PL1**	1	1	1	1	1
**CAZYme gene**	214	228	227	204	195
**% CAZome**	2.61	2.70	2.64	2.53	2.34

*Pk*, *P. kaengkrachanensis*; *Pm*, *P. mira*; *Pp*, *P. phitsanulokensis*; *Ps*, *P. silvatica*; *Pt*, *P. thailandica*.

**Table 3:** The in silico physico-chemical properties of *Planotetraspora* spp. cellulase protein predicted by ExPASSy server.

Origin	Property	Value
*P. phitsanulokensis*	Number of amino acids (AA)	461
	Molecular weight (Da)	52,821.00
	Theoretical pI	5.11
	Total number of negatively charged residues (Asp + Glu)	73
	Total number of positively charged residues (Arg + Lys)	51
	Extinction coefficient (EC)	92,040
	Instability index (II)	37.30 (Stable)
	Aliphatic index (AI)	70.91
	Grand average of hydropathicity (GRAVY)	−0.503
*P. silvatica*	Number of amino acids (AA)	461
	Molecular weight (Da)	52,941.10
	Theoretical pI	4.96
	Total number of negatively charged residues (Asp + Glu)	75
	Total number of positively charged residues (Arg + Lys)	52
	Extinction coefficient (EC)	93,530
	Instability index (II)	37.10 (Stable)
	Aliphatic index (AI)	70.69
	Grand average of hydropathicity (GRAVY)	−0.531
*P. mira*	Number of amino acids (AA)	461
	Molecular weight (Da)	52,937.14
	Theoretical pI	5.00
	Total number of negatively charged residues (Asp + Glu)	75
	Total number of positively charged residues (Arg + Lys)	53
	Extinction coefficient (EC)	93,530
	Instability index (II)	37.99 (Stable)
	Aliphatic index (AI)	71.97
	Grand average of hydropathicity (GRAVY)	−0.525
*P. thailandica*	Number of amino acids (AA)	461
	Molecular weight (Da)	52,891.07
	Theoretical pI	5.14
	Total number of negatively charged residues (Asp + Glu)	72
	Total number of positively charged residues (Arg + Lys)	52
	Extinction coefficient (EC)	93,530
	Instability index (II)	34.91 (Stable)
	Aliphatic index (AI)	68.98
	Grand average of hydropathicity (GRAVY)	−0.505
*P. kaengkrachanensis*	Number of amino acids (AA)	461
	Molecular weight (Da)	52,852.09
	Theoretical pI	5.04
	Total number of negatively charged residues (Asp + Glu)	73
	Total number of positively charged residues (Arg + Lys)	51
	Extinction coefficient (EC)	93,530
	Instability index (II)	36.50 (Stable)
	Aliphatic index (AI)	71.95
	Grand average of hydropathicity (GRAVY)	−0.478

**Table 4:** The chemical characteristics of the selected ligands for the molecular docking of the *Planotetraspora* cellulase A2 receptor.

Ligand	Pubchem CID	Molecular Formula	Molecular Weight (g/mol)
Cellobiose	10712	C_12_H_22_O_11_	342.30
Cellotetraose	439626	C_24_H_42_O_21_	666.60
Laminaribiose	439637	C_12_H_22_O_11_	342.30
Carboxymethylcellulose	24748	C_8_H_16_O_8_	240.21
Glucose	5793	C_6_H_12_O_6_	180.16
Xylose	135191	C_5_H_10_O_5_	150.13

**Table 5:** Minimum binding energy and putative cavity size from CB-DOCK web interface using Vina scores for the *Planotetraspora* cellulase A2.

Ligand	Ligand Pubchem CID	Vina Score (kJ/mol)	Cavity Size
Cellobiose	10712	−7.8	619
Cellotetraose	439626	−8.3	619
Laminaribiose	439637	−7.5	619
Carboxymethyl cellulose	24748	−5.3	1304
Glucose	5793	−6.2	1304
Xylose	135191	−5.6	1304

## Data Availability

The data used to support the findings of this study are included within the article. All data analyzed during this study (Genome assembly, GenBank assembly accession number, etc.) are available through the NCBI GenBank database and are accessible through the accession numbers listed in Table 1. Raw data are available from the corresponding author upon request.

## Supplementary Materials

The following supporting information can be downloaded at: https://www.mdpi.com/article/10.3390/genes15091202/s1, Table S1: Values of 16S rRNA, dDDH and ANI observed between different species of *Planotetraspora*; Table S2: Subsystem category distribution of whole genomes of *Planotetraspora* species annotated using RASTtk.
