# Supplementary material for: The Phylogenomic Characterization of Planotetraspora Species and Their Cellulases for Biotechnological Applications
Source: Genes (Basel). 2024 Sep 12;15(9):1202. doi: 10.3390/genes15091202 (PMC11431748; doi:10.3390/genes15091202)
Supplement: Supplementary file 1 [file genes-15-01202-s001.zip › genes-3191911-supplementary.pdf]

Table S1. Values of 16S rRNA, dDDH and ANI observed between different species of *Planotetraspora*.

	**16S rRNA (%)**					**dDDH (d_4, in %)**					**ANI (%)**				
	Pk	***Pm***	***Pp***	***Ps***	***Pt***	***Pk***	***Pm***	***Pp***	***Ps***	***Pt***	***Pk***	***Pm***	***Pp***	***Ps***	***Pt***
Pk	**100**	99.15	98.73	99.29	98.80	**100**	39.0	49.0	38.9	28.9	**100**	89.60	92.67	89.49	84.47
Pm		**100**	98.27	99.65	98.47		**100**	41.0	62.3	29.0		**100**	90.24	95.25	84.54
Pp			**100**	98.61	98.54			**100**	40.9	29.9			**100**	90.20	84.97
Ps				**100**	98.61				**100**	28.8				**100**	84.53
Pt					**100**					**100**					**100**

Pk, P. kaengkrachanensis; Pm, P. mira; Pp, P. phitsanulokensis; Ps, P. silvatica; Pt, P. thailandica

Table S2. Subsystem category distribution of whole genomes of *Planotetraspora* species annotated using RASTtk.

	**Number of predicted genes in each species**				
**Subsystem features (Functional description)**	***Pk***	***Pm***	***Pp***	***Ps***	***Pt***
**Cofactors, vitamins, prosthetic groups, pigments**	172	185	184	179	177
**Cell wall and capsule**	47	49	50	49	41
**Virulence, disease, defense**	64	58	62	60	64
**Potassium metabolism**	13	13	15	10	16
**Photosynthesis**	0	0	0	0	0
**Miscellaneous**	40	50	51	46	42
**Phages, prophages, transposable elements, plasmids**	2	4	3	6	5
**Membrane transport**	42	37	38	33	39
**Iron acquisition and metabolism**	9	9	9	10	10
**RNA metabolism**	62	61	62	61	62
**Nucleosides and nucleotides**	99	98	98	98	97
**Protein metabolism**	218	233	227	223	228
**Cell division and cell cycle**	0	0	0	0	0
**Motility and chemotaxis**	0	0	0	0	0
**Regulation and cell signaling**	14	16	15	14	14
**Secondary metabolism**	4	0	1	3	1
**DNA metabolism**	91	97	96	88	95
**Fatty acids, lipids and isoprenoids**	142	127	131	134	132
**Nitrogen metabolism**	14	17	15	16	17
**Dormancy and sporulation**	1	1	1	1	1
**Respiration**	144	146	147	137	158
**Stress response**	49	47	51	49	50
**Metabolism of aromatic compounds**	53	51	47	46	37
**Amino acids and derivatives**	349	345	347	339	329
**Sulfur metabolism**	10	10	12	9	8
**Phosphorus metabolism**	33	36	35	34	32
**Carbohydrates**	329	331	326	334	341
**Total number of genes**	2001	2021	2024	1979	1996

Pk, P. kaengkrachanensis; Pm, P. mira; Pp, P. phitsanulokensis; Ps, P. silvatica; Pt, P. thailandica
